# Improving the efficiency of testing database functionality through statistical involvement

**DOI:** 10.1186/1745-6215-16-S2-P30

**Published:** 2015-11-16

**Authors:** Gemma Clayton, Katie Pike, Rachel Nash, David Hutton, Chris Rogers

**Affiliations:** 1 Clinical Trials and Evaluation Unit, School of Clinical Sciences, University of Bristol, Bristol, UK

## Background

As a registered clinical trials unit we develop customised databases to collect and store study data and manage clinical trials; these databases need rigorous testing to ensure they function as intended, that data validation is implemented correctly and that study data extracts are complete and accurate. We describe how, with statistical involvement, the testing has been streamlined and the timelines reduced.

## Methods

The database specification, produced at the start of the database development process, details how data should be stored in the study database. Statistical programs have been written to create datasets from this specification document that are then used to test the database extracts. These datasets created are imported directly into the study databases by the IT team, thereby eliminating the need for test data to be entered manually. Once uploaded, extracts are produced and compared statistically with the original datasets. This process allows any issues, including incorrect mapping of labels to numeric values and extracting of variables under incorrect names, to be quickly identified and resolved.

## Discussion

The process for testing has evolved from a time-consuming data entry based approach to one that can be fully automated and completed in a shorter timeframe. Involvement of the statistics team has helped maximise efficiency through the use of existing specification documents for this process and ensures consistency in testing across different study databases.

## Disclaimer

This research is supported by a National Institute for Health Research (NIHR) Bristol Cardiovascular Biomedical Research Unit. The views and opinions expressed therein are those of the authors and do not necessarily reflect those of the NIHR, NHS or the Department of Health.

